# Strategy and safety at stop intersections in older adults with mild cognitive impairment and visual decline

**DOI:** 10.1016/j.trip.2023.100939

**Published:** 2023-10-18

**Authors:** Guillermo Basulto-Elias, Shauna Hallmark, Ashirwad Barnwal, Anuj Sharma, Matthew Rizzo, Jennifer Merickel

**Affiliations:** a Institute for Transportation at Iowa State University, 2711 S. Loop Drive, Suite 4700, Ames, IA 50010, USA; b University of Nebraska Medical Center, 985880 Nebraska Me, Omaha, Nebraska 68198-5800 USA

**Keywords:** Naturalistic driving study data, Intersection, Traffic safety, Older driver, Cognitive dysfunction, Mild cognitive impairment

## Abstract

This study assessed the impact of age-related cognitive and visual declines on stop-controlled intersection stopping and scanning behaviors across varying roadway, traffic, and environmental challenges.

Real-world driver data, collected from drivers’ personal vehicles using in-vehicle sensor systems, was analyzed in 68 older adults (65–90 years old) with and without mild cognitive impairment (MCI) and with a range of age-related visual declines. Driver behavior, environmental characteristics, and traffic characteristic were examined across 2,596 approaches at 173 stop-controlled intersections. A mixed-effects logistic regression modeled stopping behavior as a binary response (full stop or rolling/no-stop).

Overall, drivers who scanned more on intersection approaches (OR = 0.77) or had more visual decline (OR = 2.28) were more likely to make full stops at a stop-controlled approach. Drivers with a contrast sensitivity logMAR score > 0.8 showed the greatest probability of making a full stop compared across all drivers. Drivers without MCI were ~ 5 times more likely to come to a full stop when they scanned more (23 % versus 5 % when they scanned less) compared to drivers with MCI, who were only twice as likely to stop (14 % versus 6 % when they scanned less). Drivers were more likely to fully stop on two-lane roadways (1.5 %), during night (2.0 %), and at intersections with opposing vehicles (10.4 %).

Findings illuminate how driver strategies interact with underlying impairment. While drivers with visual decline adopt strategies that may improve safety, when drivers with MCI adopt strategies it did not result in the same degree of improvement in stopping which may result in greater risk.

## 1. Introduction

Older drivers are at risk for age-related cognitive and visual decline, which increases the risk of unsafe driving that may lead to a crash, or driving curtailment and cessation (Owsley and McGwin, 2016, Merickel et al., 2019). Roadway safety studies have shown that intersections pose challenges for older drivers due to increased demands on driver abilities that commonly decline with aging across perception, attention, motor control, executive functioning, and processing speed domains. While older driver crash risk at intersections is previously documented (Sun et al., 2018; Seacrist et al., 2020), more data are needed to determine 1) how objective driver behavior patterns and strategies, like scanning behavior at intersections, change with a cognitive and visual decline in aging, and 2) specific driver metrics that can be used to identify older drivers with elevated driving risk before a safety event occurs.

In line with this need, our study aimed to identify how age-related cognitive and visual declines impact driver behavior and strategies at stop-controlled intersections. Data inform guidelines to identify older drivers at elevated driving risk for clinical and educational interventions to preserve safety and mitigate mobility and quality of life declines that often lead to driving curtailment and cessation. Of key and novel interest was how driver strategies, like scanning or glance behavior, at intersections interact with age-related cognitive and visual declines that impact driver safety.

### 1.1. Background

Intersections require visual, attentional, motor, executive, and other functional abilities to navigate (Barnwal et al., 2022) and pose a risk to older drivers who commonly have age-related cognitive-perceptual declines (Sun et al., 2018). Analyses of the SHRP 2 naturalistic driving study (NDS) data found that older drivers (>70 years) had a greater intersection crash risk than younger drivers (Seacrist et al., 2020). The risk of a fatal intersection crash increases with age. Drivers aged 75–79 years have a 24 % increased fatal crash risk compared to those 35–74 years with an increased risk of 66–107 % for drivers aged 80 and older (Lombardi et al., 2017).

**Failure-to-yield** is a common contributing factor for older drivers involved in intersection crashes. Data (2002 to 2006) from the Fatality Analysis Reporting System (FARS) found that older drivers were more likely to be at fault in crashes at stop-controlled intersections compared to younger drivers (Sifrit et al., 2011). Specifically, drivers aged 70–79 were 2.9 times more likely, and drivers aged 80 years and older were 7.5 times more likely to be at fault than younger drivers (Braitman et al., 2007) were.

**Turning movements at intersections** can also challenge older drivers. Previous studies have found that older drivers are twice as likely to be at fault when turning compared to driving straight at a stop sign. Failure-to-yield concerns are also elevated during turning movements because older drivers may make more evaluation errors, such as not seeing an approaching vehicle (Braitman et al., 2007).

**Scanning intersections on approach** is problematic for older drivers since a wide area needs to be scanned, requiring large lateral head rotations as well as eye movements (Savage et al., 2020). Based on an evaluation using the National Motor Vehicle Crash Causation Survey (NMVCCS), the primary contributing factors at stop-controlled intersections for drivers aged 55 and older are “inadequate surveillance” and “misjudgment of gap or other’s speed” (Choi, 2010). Simulator studies have provided data that older drivers scan intersections less extensively than younger drivers, showing more fixations to their intended travel path, fewer glances to potential hazards and road markings, and smaller scan angles (Romoser et al., 2013; Savage et al., 2020; Dukic and Broberg, 2012).

**Age-related cognitive and visual dysfunction** can also complicate on-road intersection safety in older drivers and may relate to turning, failure to yield, and scanning impairments. For older drivers (≥65) compared to younger drivers, left-turn crashes at signalized intersections were more likely to be related to health and cognitive factors than intersection characteristics (Zafian et al., 2021). Another study evaluated visual search duration and angle at intersections using wearable wireless sensors (Renge et al., 2020). In this study, cognitive impairments based on Mini-Mental State Examination (MMSE) scores were correlated with smaller side-to-side visual search angles. Drivers with cognitive and visual dysfunction also exhibit more erratic braking and accelerations, even in early decline (Merickel et al., 2019).

Although concerns have been raised about older drivers’ abilities to negotiate intersections safely, older drivers may use adaptive strategies to maintain safe driving. For instance, studies have shown that drivers with visual or cognitive dysfunction drive less and may drive more often in familiar environments like residential roadways or those closer to home (Owsley and McGwin 2010; Freeman et al. 2006; Connors et al. 2017; Merickel et al., 2019). While this strategy may reduce risk, drivers (particularly those with cognitive dysfunction) may not appropriately restrict driving in high-risk environments (Mazer et al., 2021; Merickel et al., 2019).

While prior literature has identified several intersection risk factors for older drivers (e.g., turning movements, scan patterns, cognitive and visual dysfunctions), information is still lacking on the extent to which cognitive decline impacts intersection negotiation.

### 1.2. Scope

This study aimed to assess whether older drivers with age-related cognitive and visual dysfunction showed differences in stopping and scan behavior at urban, stop-controlled intersections. Stopping behavior for a set of older drivers was extracted from a NDS conducted in Omaha, Nebraska. Driver behavior was assessed by comparing drivers with a range of cognitive and visual abilities.

Driver behaviors (e.g., type of stop, glances), environmental characteristics (e.g., time of day), and traffic characteristics (e.g., presence of nearby vehicles or pedestrians) data were reduced using an expert coder for a total of 2,596 driving approaches at 182 stop-controlled intersections for 68 older drivers. A logistic regression with mixed effects was used to model stopping behavior as a binary response (full stop or rolling/no-stop). Several researchers have utilized this method to assess the impact of driver and roadway characteristics using NDS data (One-year et al., 2018; Wang and Zhou, 2018; Li et al., 2019; Chandran, 2019).

## 2. Material and methods

### 2.1. Study design

Each driver participated for two years, and naturalistic driving data were collected across two different 3-month periods separated by 12 months. Driver eligibility was assessed at study start. In-laboratory driver data were collected at the start of each study year. Drivers consented to study participation following institutional guidelines (University of Nebraska Medical Center [UNMC] IRB #217-15-FB).

### 2.2. Participant characteristics

This study enrolled 91 legally licensed, active, older drivers (aged 65–90 years) who were recruited from the community surrounding the UNMC in Omaha, Nebraska, via social media, newspaper, flyers, and talks at community organizations for older adults (Merickel et al., 2019). Of these drivers, 18 dropped out: 13 due to unwillingness to complete the study procedures, four refused to consent to share their data, and one due to recent surgery, resulting in 73 drivers. Key driver demographics, visual and cognitive data of 73 participants who had data available for analysis are summarized in Table 1.

All drivers met Nebraska licensure standards for visual acuity (better than 20/50 OU, corrected or uncorrected) and self-reported no confounding medical conditions or medication usage. Confounding medications were stimulants, narcotics, anxiolytics, anticonvulsants, antipsychotics, and other major psychoactive medications. Confounding medical conditions included dementia, sleep disorders, pulmonary disease, congestive heart failure, major psychiatric illness, vestibular disease, and current substance use. Other physical limitations (e.g., arthritis) were not excluded due to their prevalence in older populations. Drivers included in the study showed a range of age-related dysfunction (typical aging to mild cognitive impairment [MCI]) commonly seen in aging cohorts) (Ferri et al., 2005; Petersen et al. 2010).

### 2.3. Driver in-laboratory data collection

#### Demographic and Health History Data:

All drivers completed self-report questionnaires assessing demographics (age, sex, race/ethnicity, socioeconomic status [income, employment], and driving experience) and medical history (medication usage, diagnoses).

#### Vision Data:

Driver vision data were collected using ETDRS charts (full form developed in the Early Treatment Diabetic Retinopathy Study; Leder and Elman, 2018). Visual contrast sensitivity, which measures a person’s ability to see under low contrast conditions (e.g., night, poor weather), was collected at 2.5 %. Contrast sensitivity is not typically assessed for licensure and can be a more sensitive measure of visual impairment than acuity at full contrast (Johnson and Wilkinson, 2010; Wang et al., 2021). Vision scores were translated to logarithms of the minimum angle of resolution (logMAR) scales for standardized analysis. Higher logMAR scores indicate worse contrast sensitivity.

#### Cognitive Data:

Cognitive abilities were assessed using the Montreal Cognitive Assessment (MoCA). MoCA is a commonly used clinical screener for clinically significant cognitive change with established validity and reliability for detecting age-related cognitive declines and MCI. It briefly (~10 min) assesses domains that commonly decline in aging, including memory, visuospatial abilities, attention, and executive functioning (Freitas et al., 2013). A MoCA score < 26 is a standard for the cutoff to indicate a positive screen for MCI that shows good discrimination based on clinical diagnosis (Milani et al., 2018). A threshold of 26 was used in this study to provide comparability to the broader literature. Drivers who screened positive for MCI based on a MoCA < 26 were classed as “MCI.”.

### 2.4. In-vehicle data collection

A custom-built data acquisition system (DAS), developed by our group, was unobtrusively mounted on the vehicle’s windshield next to the rearview mirror in each driver’s personal vehicle at the start of each study year. The DAS included a set of sensors that collected vehicle parameters every second from on– to off-ignition. Sensors included a Global Positioning System (GPS), accelerometer, video cameras (collecting forward roadway and cabin videos along with cabin audio recordings), and other vehicle sensors that collected speed, throttle, and brake data. Drivers were instructed to drive as they typically would.

## Data processing and extraction

### 2.5. Description of intersections utilized

Potential stop-controlled intersections in Omaha were identified using coding within an R package developed by Barnwal et al. (2022). This method used driver trajectory video clips and image extraction to identify potential stop-controlled intersection locations. The GPS location of potential intersection approaches was extracted, and stop sign presence was manually confirmed. The identity of the driver (consented participant or other) was also coded. Over 750 stop-controlled intersection approaches were identified and reviewed, and those with atypical conditions were excluded (e.g., significant skew, railroad tracks nearby, brick pavement, significantly damaged pavement, more than four approaches to the intersection, or being located within a commercial area). Intersection and approach characteristics were manually coded using an interactive application developed in Shiny (Chang et al., 2015) within R and Google Streetview for the remaining 480 intersections. Finally, intersection approaches for participants’ vehicles were mapped to the identified intersection approaches, resulting in 182 stop-controlled intersections where participant data were present. A summary of intersection characteristics represented in the final set of intersections is shown in Table 2. As noted, 101 intersections were a minor road to a major road, while 81 were the intersection of two minor roads. The majority were 4-approach intersections (n = 112), with 70 having 3 approaches (T-intersection). Most had stop control on the minor approach (n = 179), while 3 were all-way stops. Several intersection countermeasures were present at a subset of the intersections, such as double stop signs, stop bars, and stop sign beacons. Since the sample size was small, countermeasures were not ultimately included in the model.

### 2.6. Selection of intersection approaches

Vehicle approaches were mapped to the corresponding intersection approaches, and the number of approaches for a particular vehicle were summarized for each approach. A total of 4,226 videos from these approaches were reviewed. In some cases, a driver traversed the same intersection approach multiple times during a given 3-month data collection period. When this occurred, to avoid oversampling, two observations were randomly selected for each intersection approach for each participant for each of the two 3-month data collection periods. Approaches where the driver was not the consented study participant were also removed.

### 2.7. Extraction of glance

Glance characteristics were extracted using in-vehicle cabin video, capturing the driver’s face, using a single coder who has significant experience coding characteristics from driver face video. Glances were coded using head position to the right or left rather than using eye movement since eye movement alone could not be detected well in the video. Glance characteristics were coded using the following convention.

- First glance location: Direction the driver visibly glanced first as they arrived at the intersection
- Right glances: Number of times a driver visibly glanced right as they arrived at the intersection
- Left glances: Number of times a driver visibly glanced left as they arrived at the intersection

Independent variables utilizing glance location are described in Table 3.

### 2.8. Type of stop

The type of stop each driver made was coded as follows:

- **Full stop:** Speed in the vicinity of the stop bar was reduced to approximately zero.
- **Rolling stop:** Clear braking was noted, and vehicle speed was greater than zero but less than five miles per hour in the vicinity of the stop bar.
- **No-stop:** Vehicle speed was greater than five miles per hour in the vicinity of the stop bar.

A description of type of stop as the dependent variable is provided in Section 3.1.

### 2.9. Traffic features

Factors that were present at the driver’s stop sign approach and could influence driver behavior were coded. Traffic features that were included in the model are described in Table 4. This included vehicles at other approaches for the intersection, pedestrians within or near the intersection, and lead vehicles (when the driver was following another vehicle). Other vehicles present at the intersection were further coded as “crossing vehicles” (presence of a vehicle on approach perpendicular to the participant’s vehicle) or “opposing vehicles” (presence of a vehicle on the approach across the intersection from the participant’s vehicle). A subjective measure was used to indicate whether presence of another vehicle(s) or pedestrian(s) was likely to have impacted the participant’s behavior. Two team members with traffic engineering expertise developed a set of logic instructions to indicate whether another vehicle or pedestrian was present but not likely to have impacted the driver (“present without effect”) or was likely to have impacted driver behavior (“present with effect”). This logic was used to categorize data. For instance, a vehicle or pedestrian crossing in front of the participant’s vehicle on stop sign approach was categorized as “present with effect.” A pedestrian walking towards the intersection but not crossing or attempting to cross would have been coded as “present without effect.”

### 2.10. Environmental features

Weather and lighting conditions were also coded. They included time of day (day, night with overhead street lighting present, night with no lighting present, and dawn/dusk). Ambient conditions were also coded as dry, light rain, and rain. Approaches with snow or adverse weather conditions were not included.

### 2.11. Final dataset post-processing

A final sample of 68 drivers across 2,596 intersection approaches and 182 stop-controlled intersections were used for analysis. Five drivers from the original sample of 73 drivers were not included because they did not traverse any of the 181 intersections that met inclusion criteria (see 2.5 Description of Intersections Utilized).

## 3. Data analysis

### Model Type and Model Selection:

Driver stops were modeled using a Bayesian implementation of a mixed-effects logistic regression to quantify the probability of a full stop at an intersection (odds ratio estimates). Random intercepts accounted for intersection- and participant-level repeated measures. Final models were built by combining data-driven and hypothesis-driven approaches. Control covariates (see below) were evaluated and selected using the leaving one out (LOO) cross-validation model selection procedure from the R package “loo” (Vehtari, et al., 2017). Different models with *a priori* interest two-way interactions were compared, and the best model according to LOO cross-validation was selected.

Models were run using R and the statistical package “brms,” (Bürkner, 2021) which uses Stan for model fitting. Each model had four Markov Chain Monte Carlo (MCMC) chains with 20,000 iterations. Half of those iterations were used as the warm-up step for the model, with a thinning rate of 8 (that is, keeping 1 out of 8 samples). Models were assessed with posterior predictive checks. All the priors used for the model were non-informative. The chain convergence was evaluated with the potential scale reduction factor (a.k.a. R-hat) and trace plots.

### Dependent Variable:

Driver stopping behavior was modeled as “full stop” or “rolling stop/no stop.” Given the low frequency of “no stops” (n = 12; 0.46 % of stops), “no stops” and “rolling stops” were combined into a single category. A total of 2,596 stops (avg. encounters per driver = 38.2) across 181 stop-controlled intersections were modeled. Full stops (n = 960), required by traffic law, were considered to be the safest action since a full stop provides a clear message to other drivers or road users of the driver’s intent, allows the driver more time to process information, and provides more reaction time for the driver and other road users. Rolling and no stops are considered less safe (n = 1,636).

### Driver Behavior Covariates of *A Priori* Interest:

Driver behavior covariates of *a priori* hypothesis interest were driver cognitive (MCI), visual (contrast sensitivity), and glance data (multiple glances, Table 3). Driver MCI was modeled as a binary variable (“yes” or “no”). Contrast sensitivity (logMAR score) was modeled as a spline to capture nonlinear relationships. Driver education programs (Muttart et al., 2011; CA, 2022; Varsity Driving Academy, 2016; DriversEdDirect, 2022; ASWDA, 2016) recommend a rule-based, left–right-left glance sequence when approaching an intersection. In line with these recommendations, glance behavior was modeled as a binary covariate indicating if the driver made multiple (“yes” or “no”) left or right glances on intersection approach. Drivers were coded as making multiple glances when they glanced **≥** 4 times to the left or right, indicating a higher level of intersection scanning. Any other combination of glances was categorized as low to normal glances. To investigate other scanning behaviors, glances were also analyzed based on 1) “first glance location” (“left” or “right”) and the number of glances (“left glances” or “right glances”). The two-way interactions considered were MCI with turning direction and number of glances, and contrast sensitivity with turning direction and number of glances. The final model includes the number of glances, the driver’s MCI class, and a control interaction of turning movement and crossing vehicle status.

### Control Covariates:

Control covariates included in the model are summarized in Table 4. Covariates were summarized in the *Data Processing and Extraction* section and were broadly selected to represent driver, environmental, traffic, and intersection characteristics that may affect driver stopping behavior. Some features had very few counts at some levels and were combined. For instance, “light rain” and “rain” were collapsed into a single variable for the covariate category “weather.” “Dawn”, “dusk”, and “night” were also collapsed into a single variable “night” for the covariate category “time of day” (TOD). Covariates included in models were selected using the LOO procedure with the exception that driver covariates of primary, *a priori* hypothesis interest (fixed effects and their two-way interactions) were maintained across all models. Final variables included in the model post-model selection are listed in **bold** in Table 4.

## 4. Results

After model selection, the final model included seven covariates that were statistically significant, with two interactions being present: (crossing vehicle status * turning movement) and (multiple glances * driver MCI). Tables 5 and 6 show the estimates and their credible intervals of the final model estimates for fixed effects (*β*), random effects (*σ*_*I*_ and *σ*_*S*_) and splines of the variables that were included in the final model (post model comparison). The estimates can also be expressed as odds ratios (OR). OR were calculated using the exponent of the estimates and credible intervals (Table 5). OR are presented for covariates that did not have interactions and are shown in Table 7. Covariates where interactions are present are better explained graphically, as noted in the following sections. The results are described in the following sections by category of covariate (i.e., driver, environmental).

### 4.1. Model results: Driver behavior

**Fig. 1.**
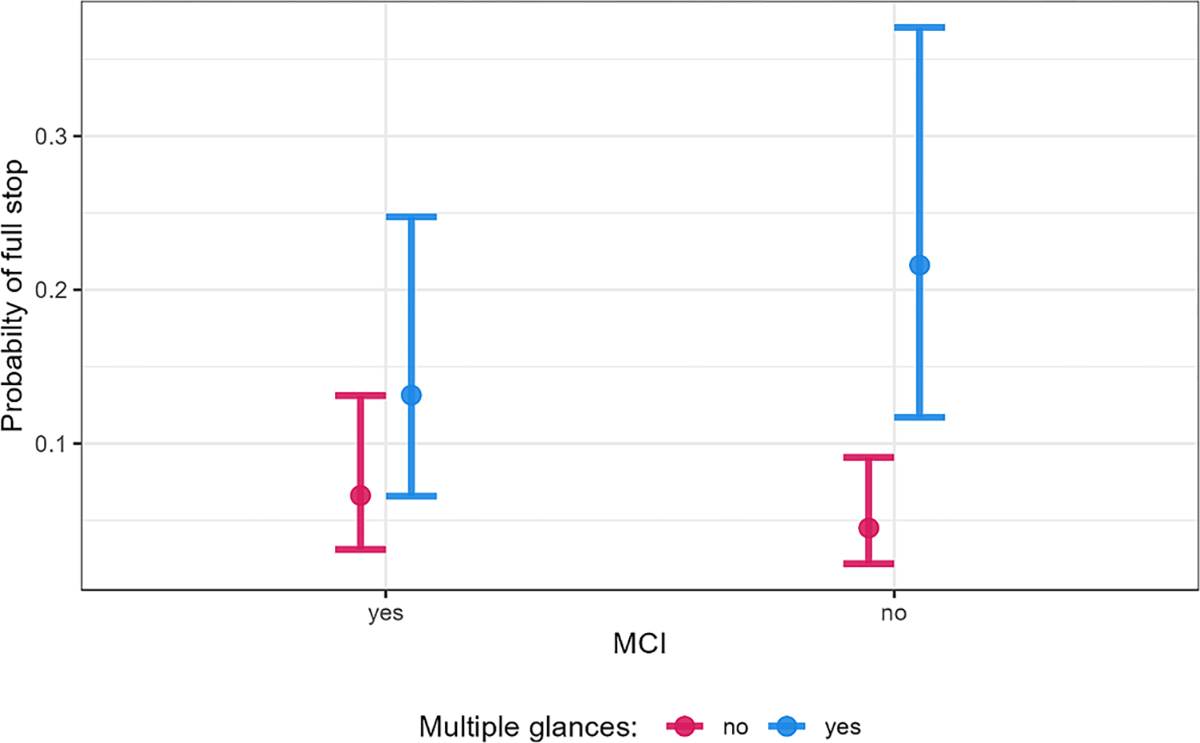
Conditional effect plot of multiple glances and driver MCI.

Overall, drivers with worse contrast sensitivity, MCI, and those who made fewer glances (“multiple glances” = “no”) were less likely to fully stop at an intersection, respectively (Table 5 and Fig. 1). Worse contrast sensitivity was associated with the largest probability of making a rolling/no-stop, compared to MCI and glance behavior. Overall, scanning more often (“multiple glances” = “yes”) improved the probability of a full stop, in line with driver education recommendations. First glance location and the overall number of left or right glances did not significantly impact stopping behavior. Driver sex and vehicle passenger had no effect on stopping behavior.

While drivers were overall more likely to stop safely when scanning multiple times on an intersection approach, increased scanning did not improve the chance of a full stop for drivers with MCI (Fig. 1). Drivers with MCI and drivers who had typical cognitive abilities showed a similar probability of making a full stop (6 % and 5 %, respectively) when scanning less on the intersection approach. However, for drivers with MCI, increased scanning resulted in a slight increase in the probability of a full stop on the intersection approach, with drivers being around twice as likely to stop (an increase from 6.6 % to 13.1 %). This was critically different from drivers who did not have MCI, who were statistically more likely to fully stop when scanning more at a magnitude of almost five times (23 %) compared to when they scanned less (5 %). This suggests that for drivers at risk of MCI, driver behavior strategies like increased scanning at an intersection may not improve safety to the same extent as when cognitive impairment is not present.

**Fig. 2.**
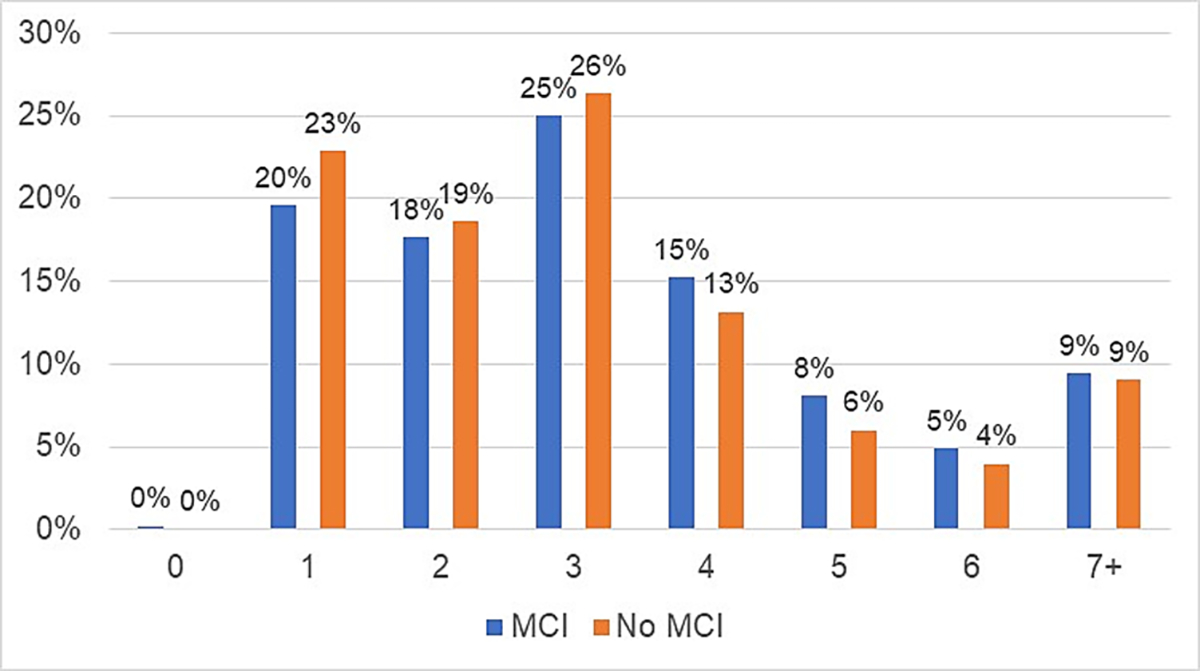
Number of glances by driver MCI.

To further investigate how driver cognitive abilities, affect driver strategy and safety, the number of glances drivers with and without MCI made on the intersection approach was examined. Fig. 2 shows the percentage of drivers by MCI category who glanced a set number of times. For instance, 23 % of drivers without MCI and 20 % of drivers with MCI glanced only 1 time as they approached the intersection. Drivers with MCI were more likely to make more than 4 glances compared to drivers without MCI. This may suggest that drivers with MCI adopt compensatory strategies, like increased scanning, when navigating challenging driving junctures like intersections without the same degree of safety improvement as seen in drivers with typical cognitive abilities.

**Fig. 3.**
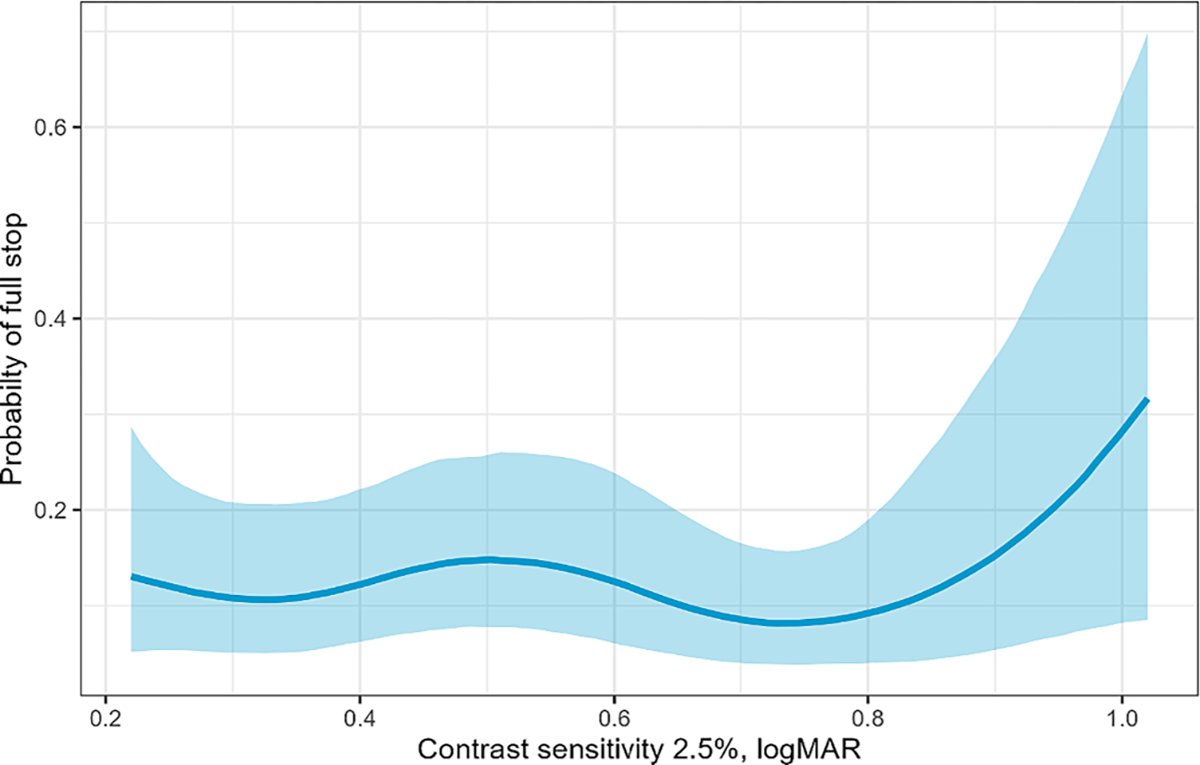
Conditional plot of contrast sensitivity and probability of full stop. Higher scores indicate worse contrast sensitivity.

Drivers with worse contrast sensitivity also showed stopping patterns that may suggest compensatory strategies to improve safety. A conditional plot showing how contrast sensitivity (spline) impacts safe stopping using a 95 % point-wise credible interval is presented in Fig. 3. Contrast sensitivity (logMAR score) was modeled as a spline to capture nonlinear relationships. Model results indicate that drivers contrast sensitivity logMAR scores of > 0.8 are more likely to make a full stop (higher logMAR scores indicate worse contrast sensitivity) and probability increases as logMAR increases. Drivers with better contrast sensitivity scores (0.2–0.8) are less likely to make a full stop (around 10 %) in general and the pattern is consistent across this range. The increasing probability of making a full stop with worsening contrast sensitivity may reflect a compensatory strategy by drivers with age-related visual decline.

### 4.2. Roadway effects

A number of covariates (Table 4) were tested to assess the impact of intersection or other roadway characteristics (i.e., roadway type, presence of channelization) on driver stopping behavior. Roadway type was the only roadway characteristic that significantly impacted the likelihood of a full stop. As shown in Table 7, drivers on a minor road intersecting with an undivided multiple-lane roadway were 0.70 times more likely to make a full stop (or 1/0.70 = 1.4 times more likely to make a rolling or no-stop) than drivers approaching a multi-lane divided roadway. Drivers on a minor street intersecting a 2-way roadway were 1.5 times more likely to make a full stop than drivers on a multi-lane divided. These results are somewhat expected since entering a multi-lane facility presents a more complicated roadway scenario and typically more conflicting traffic than a 2-lane roadway. However, surrounding traffic was a significant covariate as noted below. As a result, the presence of conflicting vehicles may be more likely to impact driver behavior than the roadway scenario.

### 4.3. Environmental effects

Several environmental characteristics (i.e., time of day), as shown in Table 4, were tested. As shown in Table 7, TOD was the only environmental characteristic that was statistically significant with drivers being 2.0 times more likely to make a full stop at nighttime than during the daytime. This may result from drivers adjusting their behaviors to account for reduced visibility.

### 4.4. Traffic effects

**Fig. 4.**
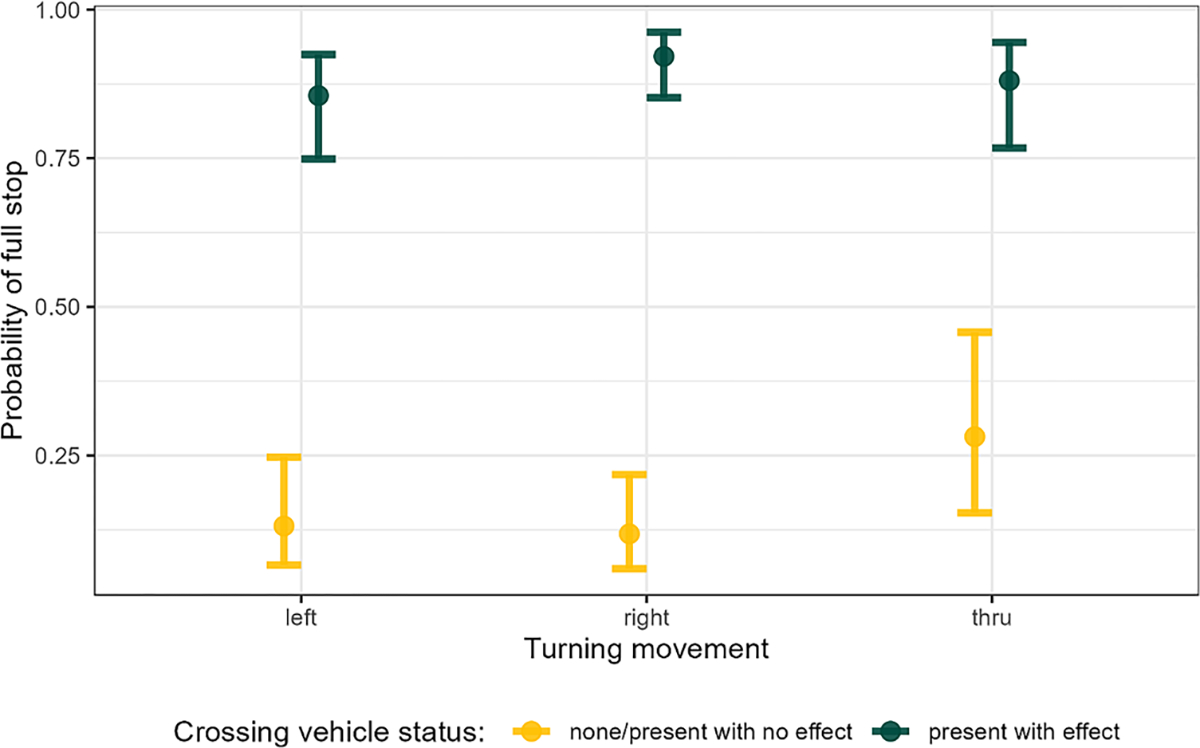
Interaction of crossing vehicle status and turning movement on full stopping.

The impact of surrounding traffic was assessed using several co-variates as shown in Table 4. As noted in Table 7, a driver was 10.4 times more likely to make a full stop when a vehicle was present on the opposing approach than when no vehicle was present. Turning movement and the presence of vehicles on the opposing approach were also statistically significant. Since an interaction was present between the two variables, they are more easily explained using a graphical representation of the interaction, as shown in Fig. 4. As noted, drivers were much more likely to make a full stop overall when crossing vehicles were present (90 % for turning right, 85 % for turning left, and 88 % for a through movement). When no crossing vehicles were present, drivers were the most likely to stop (around 15 %) when making a through movement (compared to right and left turns, which had similar probabilities (around 10 %).

## 5. Discussion and conclusions

### 5.1. Conclusions

The goal of this paper was to extend prior literature on aging and driving through objective and systematic assessments of how age-related driver cognitive and visual declines impact driver strategies and safety at intersections, with the goal of improving early identification of driver safety changes that may lead to driving curtailment or cessation, reduced mobility, and quality of life declines. The study used naturalistic driving data collected from older drivers with a range of cognitive and visual abilities. Methods permitted collection of a driver’s real-time scanning and stopping behavior at high risk driving junctures like intersections and are broadly generalizable to future studies.

Results reveal that older drivers engage in specific, quantifiable behaviors that may reflect driver decision-making and awareness. Older driver scanning behavior, independent of driver abilities, affected safety, with drivers who scanned more showing higher probabilities of full stops. This result supports current driver education recommendations and could be incorporated into older driver education programs aimed at preserving safety and mobility.

Drivers with MCI may adopt compensatory strategies to improve safety, like scanning more on intersection approaches. However, these strategies may not mitigate underlying driver safety decrements from cognitive decline, consistent with the known effects of age-related cognitive decline on executive functioning, processing, attention, and other cognitive domains. Increased scanning behavior at intersections may also reflect uncertainty and/or difficulty with decision-making and information processing that can commonly occur with cognitive decline and MCI. Results suggest that MCI presents a key risk in driving that is not be fully mitigated by driver strategy. Safety changes observed in this study occurred early in cognitive decline, before dementia onsets. None of the drivers in this sample had dementia, suggesting that changes in driver behavior may provide an index of early cognitive decline for clinical intervention.

Drivers with age-related visual declines also appear to adopt compensatory strategies, like being more likely to stop at intersections. Further research is needed to determine if these strategies improve safety; however, they may reflect driver awareness of impairment. Results provide specific guidelines for future research to assess likelihood of changes in driver safety behavior from visual decline that can be validated in a larger population. Data suggests that a logMAR score of > 0.8 for contrast sensitivity may index driver safety changes from visual decline. The use of contrast sensitivity to quantify visual abilities provides a sensitive index of age-related visual decline and driver safety impacts, even in older driver populations who meet state licensure standards.

Other findings suggest intersection, environmental, and traffic characteristics impacted stopping behavior. Drivers were more likely to stop when intersecting a minor 2-lane roadway than when intersecting a multi-lane roadway. This result was unexpected since entering a multi-lane roadway presents a more complicated scenario. However, drivers were highly likely to stop when vehicles were present on opposing intersection approaches regardless of the roadway type. As a result, the presence of other vehicles impacts behavior more than roadway type. Only a few intersection characteristics were relevant in these data to modifying stopping behavior for older driving populations, which was unexpected. As a result, no conclusions can be made about roadway design features that may challenge older drivers based on these study results. Older drivers were more likely to stop during the nighttime, which may indicate a need to increase intersection scanning due to reduced visibility.

### 5.2. Discussion

Passive monitoring of driver behavior provides novel insights into how age-related decline impacts driver strategy and safety; it demonstrates that early intervention preserves mobility and quality of life across the lifespan. Results underscore that driver abilities, cognitive and visual, not age are primary factors when considering on-road safety. How a driver’s abilities are affected by age and MCI critically impact the driver’s adoption of safety strategies. Data from remotely deployed sensors may also be able to detect behavioral changes that index worsening decline, like MCI and risk for dementia, or safety risk for intervention. By linking driver abilities to behavior, results inform the development of targeted interventions to improve safety, mobility, and quality of life in aging.

Results can also inform older driver education training. For instance, education could include specific recommendations about scanning for hazards and understanding challenges in low visibility situations. Additionally, results suggest development of personalized driver safety trainings/interventions/evaluations for each driver’s unique clinical profile can be utilized to improve driving outcomes.

While no specific roadway features were noted as problematic, results still have implications for roadway-based interventions. As noted, drivers with worse contrast sensitivity were more likely to stop. This suggests drivers are adjusting their behavior to compensate for reduced visibility. In general, older drivers were more likely to stop during the nighttime than daytime also suggesting compensation for reduced visibility. Both outcomes suggest traffic engineering strategies, such as more visible pavement markings and signs as well as overhead intersection lighting; can be effective in providing visual clues to drivers with visual decline.

### 5.3. Limitations

While this study offers several promising results, admittedly there are limitations which future research can expand upon. Further investigation is needed to determine how driver safety behaviors in more broadly defined contexts are affected by age-related declines to comprehensively assess driver risk and the impact of these behaviors on safety outcomes (e.g., crashes) and the driver (e.g., risk of driving curtailment). Future research may also investigate how driver safety behaviors link to awareness of impairment to more objectively assess driver decision-making in high-risk driving contexts. Investigating further how visual and cognitive impairments interact and vary within a single driver and across roadway contexts may provide key insight for developing improved models of older driver safety and for early detection of risk across complex, individually variable factors. Driver safety outcomes may also be linked to health and quality of life outcomes to more objectively assess how driver safety decrements impact driver outcomes. A broader population of drivers, including those with early dementia, may improve recommendations for driver safety in aging.

## Figures and Tables

**Table 1:** Summary of Participant Characteristics.

Variable	Range or counts	Average	Std. dev.

Age (years)	Range: 65–90	75.4	6.45
Sex	Female = 33		
	Male = 40		
Race	White = 70		
	African American = 1		
	American Indian = 1		
	Other = 1		
Income	$0-$500,000 Not indicated = 9	$55,362	$62,854
Employment	Employed = 22		
	Retired = 35		
	Unemployed = 13		
	Not indicated = 3		
Driving Experience (years)	Range: 40–76 Not indicated = 1	58.3	8.13
Weekly Driving	30–300 miles (48–483 km) Not indicated = 1	102 miles (164 km)	60.3 miles (97 km)
MoCA score	17–30	25.8	2.54
Contrast sensitivity 2.5 % (logMAR)	0.22–1.02	0.494	0.162

**Table 2:** Summary of Intersection Characteristics.

Characteristic	Categories	Count	Percent

Intersection configuration	Major/minor	101	55.5 %
	Minor/minor	81	44.5 %
Intersection type	T-intersection	70	38.5 %
	4 approaches	112	61.5 %
Median on major approach	Divided curb	15	8.2 %
	Divided painted	38	20.9 %
	Undivided	129	70.9 %
Stop control	All-way	3	1.6 %
	Minor approaches	179	98.4 %
Intersection countermeasures	Double stop sign*	2	1.1 %
	Stop bar	22	12.1 %
	Stop sign beacon	1	0.5 %

* Two stop signs are placed at the same approach for increased conspicuity.

**Table 3:** Driver Behavior Covariates of *A Priori* Interest were maintained in models throughout model selection and included in the final model.

Variable	Value	Count	Percent

Categorical Covariates			
First glance location	Left	1916	73.81 %
	Right	677	26.07 %
	None	3	0.12 %
Multiple glances	No	1686	64.95 %
	Yes	910	35.05 %
MCI	Yes	1366	52.62 %
	No	1230	47.38 %
Variable	Avg.	Std. Dev.	Range
Numeric Covariates			
Right glances	1.48	1.55	0 – 19
Left glances	2.00	1.56	0 – 21
Cs 2.5 % logMAR	0.5841	0.1699	0.22 – 1.02

**Table 4:** Control covariates used in model selection. Covariates were selected using the LOO procedure. Those included in the final model are in bold.

Variable	Value	Count	Percent

Driver Control Covariates			
Sex	Female	1224	47.15
	Male	1372	52.85
Vehicle Passenger Present	No	1889	72.77
	Yes	707	27.23
Environmental Control Covariates			
Time of Day	Day	2421	93.26
	Night/dawn/dusk	175	6.74
Weather	Dry	2437	93.87
	Rain/light rain	159	6.12
Traffic Control Covariates			
Lead vehicle status	None/present with no effect#	2305	88.79
	Present with effect#	291	11.21
Crossing vehicle status	None/present with no effect#	1757	67.68
	Present with effect#	839	32.32
Opposing vehicle status	None/present with no effect#	2569	98.96
	Present with effect#	27	1.04
Crossing pedestrian status	None/present with no effect#	2550	98.23
	Present with effect#	46	1.77
Turning movement	Left	774	29.82
	Right	1242	47.84
	Thru	580	22.34
Intersection Control Covariates			
Stop control type	1-way	980	37.75
	2-way	1616	62.25
Road type	Multi-lane divided	279	10.75
	Multi-lane undivided	378	14.56
	Two-lane	1939	74.69
Channelization minor	None	2533	97.57
	Yes	63	2.43
Stop bar	None present	2087	80.39
	Present	509	19.61

**Table 5:** Final Model Variables and Fixed Effect Estimates.

Term	Estimate	Lower 95 % CI	Upper 95 % CI

Intercept	− 2.07	− 2.76	− 1.38
Driver Behavior Covariates of A Priori Interest			
MCI: no	0.61	0.14	1.09
Multiple glances: yes	0.77	0.39	1.13
MCI: no	1.01	0.49	1.54
Multiple glances: yes			
Contrast sensitivity 2.5 % logMAR (spline term)	2.28	− 4.59	8.33
Environmental Control Covariates			
TOD: night/dawn/dusk (base: day)	0.70	0.22	1.17
Traffic Control Covariates			
Crossing vehicle status: present with effect (base: present without effect/not present)	3.67	3.20	4.16
Opposing vehicle status: present with effect (base: present without effect/not present)	2.34	1.24	3.46
Turning movement: thru (base: left) Crossing vehicle status: present with effect (base: present without effect/not present)	− 0.73	− 1.43	− 0.05
Turning movement: right (base: left) Crossing vehicle status: present with effect (base: present without effect/not present)	0.80	0.18	1.44
Turning movement: right (base: left)	− 0.12	− 0.55	0.32
Turning movement: thru (base: left)	0.95	0.52	1.39
Intersection Control Covariates			
Road type: multi-lane undivided (base: multi-lane divided)	− 0.36	− 1.02	0.31
Road type: Two-lane (base: multi-lane divided)	0.44	− 0.12	0.98

**Table 6:** Final model random effect estimates.

Term (Std. Dev. Parameter)	Estimate	Lower 95 % CI	Upper 95 % CI

Intersection	0.423	0.09	0.691
Participant	0.655	0.403	0.962

**Table 7:** Odds ratios of fixed effects that do not interact with other variables.

Term	Estimate	Lower 95 % CI	Upper 95 % CI

Intercept	0.127	0.063	0.251
TOD: night/dawn/dusk (base: day)	2.010	1.247	3.209
Opposing vehicle status: present with effect (base: present without effect/not present)	10.423	3.463	31.722
Road type: multi-lane undivided (base: multi-lane divided)	0.698	0.359	1.368
Road type: Two-lane (base: multi-lane divided)	1.545	0.891	2.667

## Data Availability

The authors do not have permission to share data.
